# Shared taxa but distinct communities: within-individual comparisons of oral, nasal, and urinary microbiomes in asymptomatic “healthy” females

**DOI:** 10.3389/frmbi.2026.1691965

**Published:** 2026-03-09

**Authors:** Sandra Jablonska, Alex Kula, Catherine Putonti

**Affiliations:** 1 Bioinformatics Program, Loyola University Chicago, Chicago, IL, United States; 2 Department of Biology, Loyola University Chicago, Chicago, IL, United States

**Keywords:** 16S rRNA sequencing, healthy human microbiome, nasal microbiome, oral microbiome, urinary microbiome

## Abstract

Although microbial community compositions vary throughout the healthy human body, some taxa can be found to reside in multiple anatomical sites. Moreover, some microbiota that have been found to be interconnected, e.g., the gastrointestinal tract and the oral cavity, the female urinary tract and the vagina, the nose (specifically the nares) and the skin. Previously, the urinary microbiome has only been compared to proximal sites; however, several species that inhabit the asymptomatic female urinary tract are also found in distant communities, e.g., *Streptococcus mitis*, also found in the oral cavity, and *Staphylococcus epidermidis*, also found in the nares. This prompted our investigation of communities of the oral cavity, nares, and urinary tract, collected from the same individual. In this study, we profiled the oral, nasal, and urinary microbiomes of 26 self-identified “healthy” female participants using 16S rRNA amplicon sequencing. The urinary microbiome was found to be distinct from the oral and nasal communities. Nevertheless, *Actinomyces*, *Corynebacterium*, *Escherichia* + *Shigella*, *Lawsonella*, *Staphylococcus*, and *Streptococcus* were found to be present within communities of all three anatomical sites. Genera predominant within the oral and nasal communities were often low-abundant taxa within the urinary microbiome. To date, comparisons of the urinary microbiome to microbiomes of other anatomical sites is limited. The distinction between the urinary microbiome and that of the more distant oral and nasal site highlights the role that the environment plays in shaping these communities.

## Introduction

The human microbiota plays a critical role in maintaining host health, modulating immune responses, and preventing pathogenic colonization (Belkaid and Hand, 2014; Gilbert et al., 2018). Distinct microbial community composition, shaped by anatomical location and physiological conditions, are increasingly recognized as key contributors to both health and disease (Costello et al., 2009; Human Microbiome Project Consortium, 2012). Thus each body site often harbors a unique microbial community shaped by its local environment and immune landscape (Human Microbiome Project Consortium, 2012; Ding and Schloss, 2014). For instance, the oral cavity of asymptomatic individuals is typically dominated by species of *Streptococcus*, *Veillonella*, *Actinomyces*, and *Neisseria* (Bik et al., 2010; Sharma et al., 2018). The nasal cavity often contains *Staphylococcus* as well as *Corynebacterium* and *Dolosigranulum*, taxa associated with respiratory homeostasis and pathogen defense (Bassis et al., 2014). In the urinary tract, particularly in asymptomatic females, *Lactobacillus* species predominate, maintaining a low-pH environment protective against uropathogens (Al et al., 2025), although species of *Streptococcus*, *Actinomyces*, *Staphylococcus*, and *Corynebacterium* have also been found in abundance (Price et al., 2020a; Du et al., 2024).

The microbiota of a given anatomical site does not persist in isolation. External factors can isolate or connect communities within the body. For instance, the mouth is generally considered of consisting of three distinct environments [(Eren et al., 2014) and see review by Baker et al. (Baker et al., 2024)]. Likewise, paired nasal and nasopharyngeal samples of 100 individuals showed that even these relatively proximal sites vary (De Boeck et al., 2017). While subsequent studies found associations between nasal and nasopharyngeal communities (Konovalovas et al., 2024), the microbiome of the lungs more closely resembles that of the oral microbiome, and not the nasal microbiome, within healthy individuals (Bassis et al., 2015). As another example, studies of paired urinary and vaginal microbiomes have found the two communities to be interconnected (Thomas-White K. et al., 2018; Komesu et al., 2020) with subsequent evidence of shared strains (Atkins et al., 2024; Appleberry et al., 2025). More distant anatomical sites can also be connected, perhaps the best studied is that of the oral and the gut microbiome, a.k.a. the oral-gut axis, [(see review by Kunath et al. (Kunath et al., 2024)].

In comparison to other anatomical sites of the human body, the urinary microbiota is less well characterized, as its investigation was delayed due to the belief that the urinary tract of asymptomatic individuals was sterile (Thomas-White et al., 2016). Given the fact that some of the same species of bacteria are found in the asymptomatic female urinary tract and the oral cavity, e.g., *Streptococcus mitis* (Mores et al., 2021), and the nasal cavity, e.g., *S. epidermidis* (Price et al., 2020b), we were interested to compare the microbiomes of these sites. While some studies have collected paired samples from these anatomical sites, they focused on comparing samples from asymptomatic populations to those with a disease/symptom. For instance, Maciniak et al. compared the urinary and oral microbiomes of females with/without breast cancer and Liao et al. compared the oral and nasal microbiomes of individuals with nasopharyngeal carcinomas (Liao et al., 2024; Marciniak et al., 2025). However, some studies have compared the nasal and oral/buccal microbiomes of the same individuals, including both healthy individuals (Zhou et al., 2013) as well as those with Parkinson’s (Pereira et al., 2017). While connection between the urinary tract and the gut (Thänert et al., 2019; Worby et al., 2022) and the vaginal (Thomas-White K. et al., 2018; Komesu et al., 2020; Atkins et al., 2024) communities have previously been considered, more distant sites have not. The objective of this study was to characterize and compare the oral and nasal microbiomes to the urinary microbiome in asymptomatic “healthy” adult females.

## Materials and methods

### Sample collection

Oral, nasal, and urine samples were collected from 26 participants as part of an institutional-review board (IRB) approved study (Loyola University Chicago, IRB no. 3603). Written consent was obtained from all participants. Biological females were enrolled in the study if they were between the ages of 18 and 25, self-identified as “healthy” at the time of participation, and had not taken antibiotics in the past 6 months. Buccal and nasal samples were taken using swabs (BD BBL Culture Swab) after instruction and under the supervision of a researcher. Each participant was instructed to rub the cotton swab (BD BBL CultureSwab) on their inner cheek for 30 seconds, after which the swab was inserted into the provided storage solution. Similarly, for the nasal swab, each participant was instructed to rub the cotton swab (BD BBL CultureSwab) on the lower inside of the nostril for 30 seconds; it too was then inserted into the provided storage solution (Amies media). Urine samples were collected at home by participants in a supplied sterile urine collection cup. Participants were instructed on how to collect these samples, and they were asked to collect a mid-stream voided sample of their first morning urination and bring in the sample the same day as collected.

### DNA extraction and 16S rRNA sequencing

The DNA extraction process was carried out as follows. For oral and nasal swab samples, the swab was transferred to a ZymoBIOMICS DNA Microprep kit lysis tube containing 750 µl of ZymoBIOMICS Lysis Solution. This mixture was subjected to 45 minutes of beating using a Disruptor Genie, with the duration of disruption determined in consultation with Zymo Research for the specific model of cell disruptor used. After beating, the tubes were immediately stored at -80 °C until samples from all twenty-six participants were collected. For urine samples, the collected urine was aliquoted into cryotubes, adding Assay Assure (SierraMolecular) (10% by volume), and stored at -80 °C. Prior to DNA extraction, urine samples were thawed at room temperature whereupon they were then pelleted (6.8 ml/sample) for extraction.

All DNA extractions were performed using the ZymoBIOMICS DNA Microprep kit. DNA was extracted from the processed oral and nasal samples following the manufacturer’s instructions for low biomass samples while urine samples followed the urine sample protocol found in the manufacturer’s protocol appendix B. In parallel there were two sets of negative controls (nuclease-free water) extracted for each anatomical site. All extracted samples were quantified using the Qubit fluorometer (Invitrogen).

If samples possessed quantifiable DNA, they were sent for sequencing at SeqCenter (Pittsburgh, PA USA). There, samples were prepared using Zymo Research’s Quick-16S kit with phased primers targeting the V3-V4 regions. The specific primer sequences used were 341f (5’-CCTACGGGDGGCWGCAG-3’ and 5’-CCTAYGGGGYGCWGCAG-3’) and 806r (5’-GACTACNVGGGTMTCTAATCC-3’). Following clean up and normalization, samples were sequenced on P1 600cyc NextSeq2000 Flowcell and generated 2x301 bp paired end reads.

### Sequence analysis

Paired-end FASTQ files were imported into R (v4.3.3) for microbial community analysis. Primer sequences were removed using the Cutadapt (Martin, 2011) for the V3–V4 primers 5’-CCTAYGGGNBGCWGCAG-3’ and 5’-GACTACNVGGGTMTCTAATCC-3’. These primers were trimmed from all reads prior to downstream analysis. Initial quality assessment was performed using FastQC (Andrews, 2010) to evaluate the overall base quality, the presence of adapters, and any other sequence artifacts. Afterwards, amplicon sequence variant (ASV) inference was carried out using the DADA2 package (v1.30.0) in R (Callahan et al., 2016). Reads were filtered and trimmed with truncation lengths of 275 and 250 bases for forward and reverse reads, respectively. Additional quality filtering parameters included the removal of reads with ambiguous bases (maxN = 0), filtering based on expected error rates [maxEE = c(3,3)], and truncation at the first base with a quality score below 2 (truncQ = 2). PhiX sequences were also removed. Error rates were learned from the filtered sequences and applied to infer ASVs from each sample. Forward and reverse reads were dereplicated and denoised using the dada algorithm and subsequently merged. Chimeric sequences were removed using the consensus method in the removeBimeraDenovo function.

Taxonomy was assigned using the SILVA v138.2 reference database (Quast et al., 2013) with the assignTaxonomy function. Classification was performed at the genus level using the silva_nr99_v138.2_toGenus_trainset. This allowed for robust and reproducible taxonomic labeling of ASVs. To control for processing-related contaminants, a negative control sample was included for each anatomical site. The relative abundance of taxa in each control was subtracted from the corresponding site’s samples, with any resulting negative values set to zero. The final sequence table was then converted into a phyloseq object and agglomerated at the genus level.

Relative abundances were calculated and visualized using ggplot2 v.3.5.1 (Wickham, 2024). The top 20 most abundant genera across all samples were retained for visualization. Taxa not among the top 20 were collapsed into a category labeled “Other,” which was consistently assigned a dark grey color. Unclassified taxa (“NA”) were labeled and displayed in light gray. Missing samples were visually retained in the graphs with a value of zero reads.

For additional analyses focusing on the genera detected across all three anatomical sites, mean and median relative abundances were calculated for each genus within each anatomical site to assess dominance patterns and distributional skew. Prevalence was defined as the proportion of samples within a given site in which a genus was detected (relative abundance > 0).

### Microbiome statistics

Diversity analyses were conducted using alpha and beta diversity metrics using the vegan R package (Oksanen, 2025). Shannon and Simpson indices were used to assess within-sample diversity. Differences in the relative abundance of all detected genera across microbiome types were assessed using the Kruskal-Wallis test. P-values were adjusted for multiple comparisons using the Benjamini-Hochberg procedure. All statistical analyses were performed in R.

The Principal Coordinates Analysis (PCoA) was performed based on Bray-Curtis dissimilarity of genus-level relative abundance data. Relative abundance tables were prepared for each sample site, merged into a unified matrix, and filtered to exclude samples with zero abundance. Bray-Curtis dissimilarity was computed using the vegdist() function from the vegan R package (Oksanen, 2025). PCoA was conducted using cmdscale() in base R. The first two PCoA axes were used for visualization. A 2D scatter plot was created using ggplot2 (Wickham, 2024) where each point represents a sample, colored by isolation source. 95% confidence ellipses around each group were computed using stat_ellipse(type = “norm”, level = 0.95) to represent within-group variation. PERMANOVA (adonis2() from vegan) was run with 999 permutations. Multiple comparisons were adjusted using FDR corrections. Additionally, Analysis of Similarities (ANOSIM) using the anosim() function from the vegan R package was used to return an R statistics representing degrees of separation between anatomical groups.

## Results

**Figure 1:**
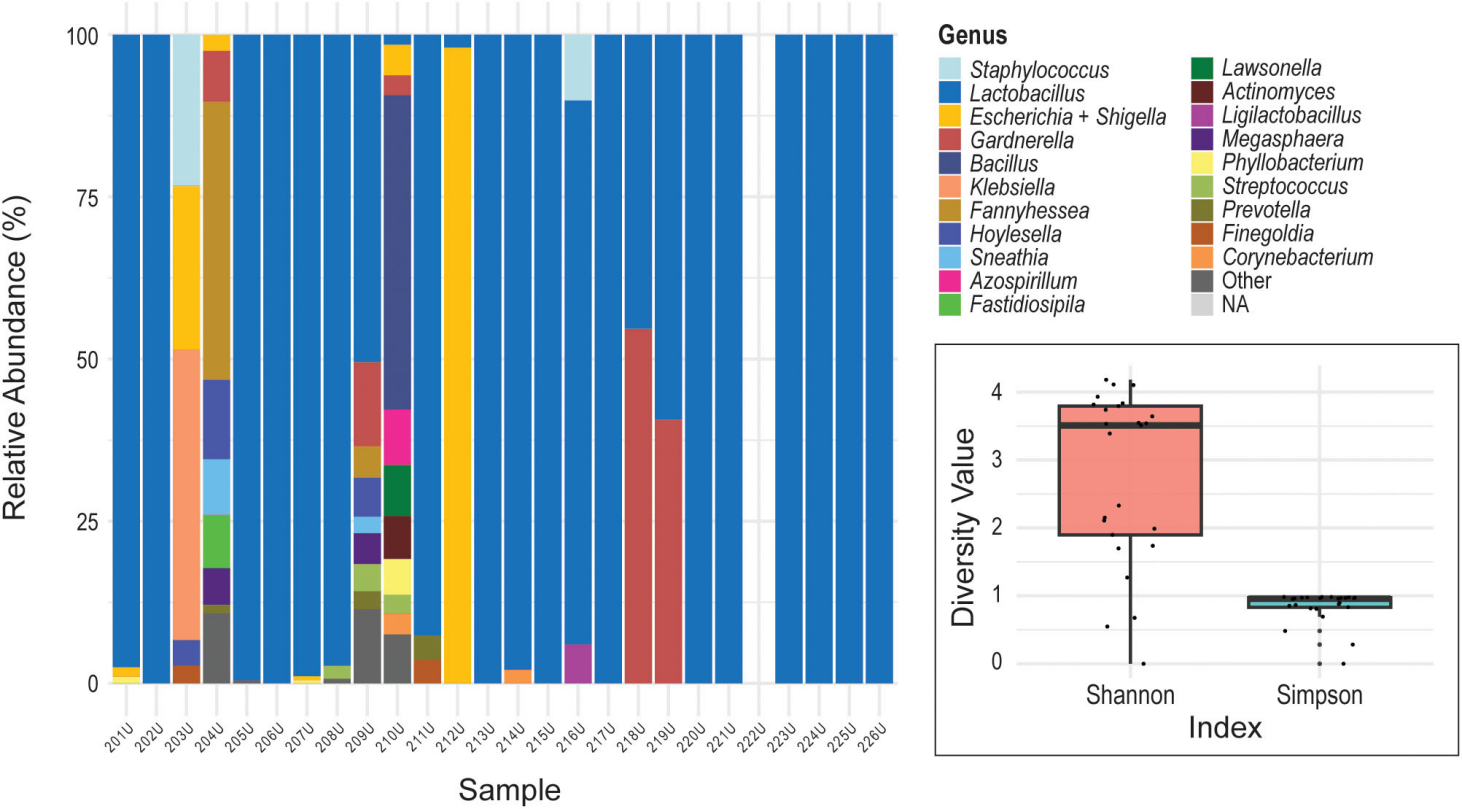
Urinary microbiome profiles. The stacked bar chart displays the relative abundance of bacterial genera in the voided urine samples (y-axis) collected from female participants (x-axis). (Participants are listed in the same order for this and subsequent figures.) Each color represents a different bacterial genus. Insert: Shannon Diversity Index and Simpson Diversity Index for the urinary microbiome. The y-axis represents the diversity value for each metric. The colored boxes represent the interquartile range (IQR; 25th to 75th percentiles) for each diversity metric. The horizontal line inside each box indicates the median value. Individual black dots represent the diversity index measurements for individual samples.

**Figure 2:**
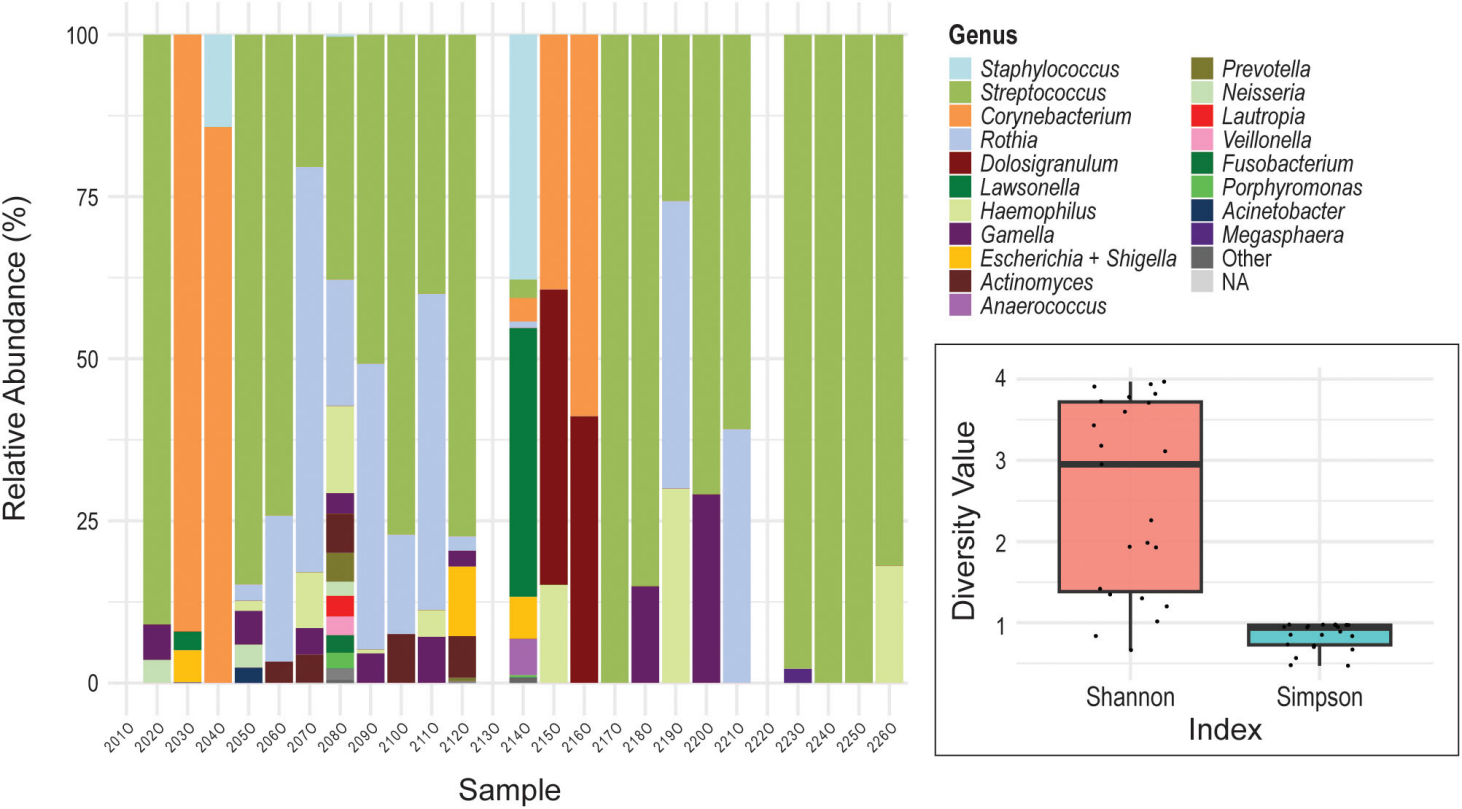
Oral microbiome profiles. The stacked bar chart displays the relative abundance of bacterial genera in the buccal swab samples (y-axis) collected from female participants (x-axis). Each color represents a different bacterial genus. Insert: Shannon Diversity Index and Simpson Diversity for the oral microbiome. The y-axis represents the diversity value for each metric. The colored boxes represent the interquartile range (IQR; 25th to 75th percentiles) for each diversity metric. The horizontal line inside each box indicates the median value. Individual black dots represent the diversity index measurements for individual samples.

**Figure 3:**
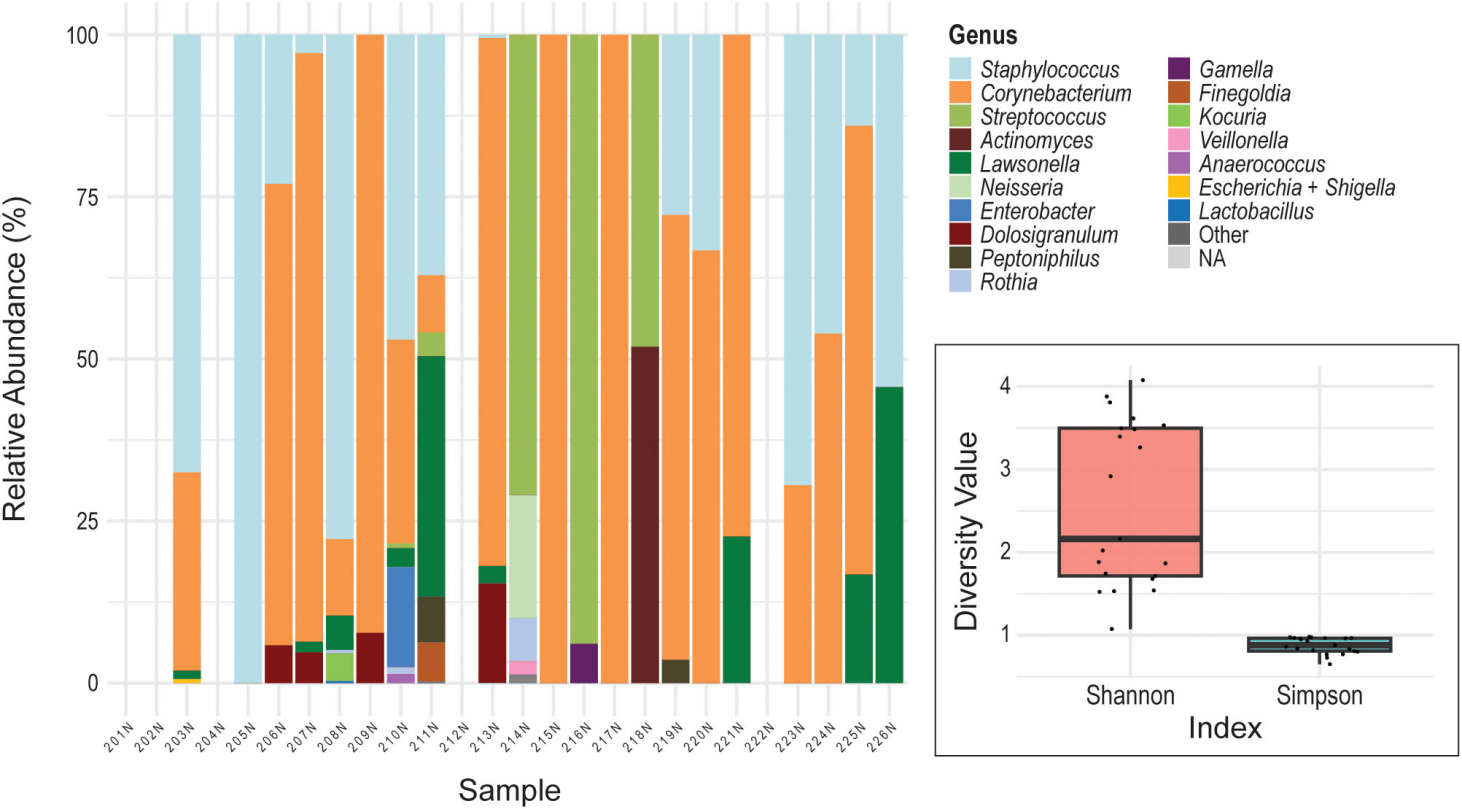
Nasal microbiome profiles. The stacked bar chart displays the relative abundance of bacterial genera in the interior nares swab samples (y-axis) collected from female participants (x-axis). Each color represents a different bacterial genus. Insert: Shannon Diversity Index and Simpson Diversity Index for the nasal microbiome. The y-axis represents the diversity value for each metric. The colored boxes represent the interquartile range (IQR; 25th to 75th percentiles) for each diversity metric. The horizontal line inside each box indicates the median value. Individual black dots represent the diversity index measurements for individual samples.

Out of the 78 samples collected, 16S rRNA metagenomic sequencing of the V3-V4 regions was successfully conducted for 23 oral samples, 21 nasal samples, and 24 urine samples. The remaining samples had low or no DNA and did not produce sufficient read depth. Details regarding the sequencing data produced can be found in **Supplementary Table S1**. On average, each sample produced 283,926 ± 84,427 reads. Reads were processed and assigned to taxonomic lineages using DADA2 and the SILVA database, respectively. While presented in detail within the Methods, raw reads were first quality controlled and amplicon sequence variant (ASV) inference was conducted. Taxonomy was then assigned to ASVs, and for each sample, decontamination was conducted using sequenced negative controls, and the relative abundance of each genus was computed. **Figures 1**–**3** present visualization of the relative abundances for the urinary, oral, and nasal samples in turn; any blank columns in relation to individual participant means that DNA was too low for sequencing or reads were filtered out during the decontamination step.

### The asymptomatic female urinary microbiome

The urinary microbiome was predominated by the genus *Lactobacillus*. In 21 out of 25 samples, *Lactobacillus* was detected, and in 11 of those, it was the only genus observed among the top 20 taxa (**Figure 1**). Other genera, including the SILVA database’s designation “Escherichia-Shigella” (representative of species belonging to the genera *Escherichia* and *Shigella* [henceforth referred to as “*Escherichia* + *Shigella*”]), *Gardnerella*, and *Bacillus*, appeared in low relative abundance for most individuals. Four samples, U203, U204, U209, and U210, exhibited mixed urotypes, i.e., the community was not dominated by a single genus. This variation in complexity is reflected in alpha diversity metrics (**Figure 1**). Here we have considered both the Shannon Diversity Index and the Simpson Diversity Index. While the Shannon Diversity Index is more sensitive to rare taxa, the Simpson Diversity Index is weighted toward dominant taxa and therefore highlights differences in community dominance. As shown in the insert in **Figure 1**, the Shannon Diversity Index values for the urinary microbiome samples ranged from below 2 to nearly 4, with higher values indicating greater richness and evenness. Conversely, the Simpson Diversity Index values were consistently high (near 1), consistent with the predominance of one or two taxa in most samples.

### The asymptomatic female oral microbiome

As shown in **Figure 2**, the oral microbiome was primarily composed of the genera *Streptococcus* and *Rothia*. *Streptococcus* was present in 17 out of 23 samples, and *Rothia* was detected in 11. While most samples exhibited mixed profiles containing two or more genera, samples O217, O224, and O225 were monodominated by a single genus. Additional genera frequently observed across the oral samples included *Corynebacterium*, *Haemophilus*, *Gemella*, *Lawsonella*, and *Dolosigranulum*. Alpha diversity metrics indicated moderate to high diversity within the oral microbiomes (**Figure 2**). The Shannon Diversity Index analysis showed a median value near 3, reflecting moderate richness and evenness across most samples. Simpson Diversity Index values were generally close to 1, suggesting a high degree of dominance by one or a few taxa in many samples. These metrics indicate that while many oral communities contained multiple taxa, they were often unevenly distributed.

### The asymptomatic female nasal microbiome

Among the nasal samples, only two (N205 and N217) were monodominated by a single genus, while the remaining samples exhibited mixed communities composed of two or more genera (**Figure 3**). The most observed genera were *Corynebacterium*, detected in 16 out of 21 samples, and *Staphylococcus*, found in 14 out of 21. *Streptococcus* and *Lawsonella* were present in 4 and 9 samples, respectively. Alpha diversity analysis indicated moderate diversity across the nasal samples (**Figure 3**). The Shannon Diversity Index analysis showed a median value of approximately 2.2 to 2.3, reflecting moderate richness and evenness. Simpson Diversity Index values were clustered near 1, consistent with the dominance of one or a few genera in most samples and relatively low community evenness.

### Comparison of microbial communities across sampled sites

**Figure 4:**
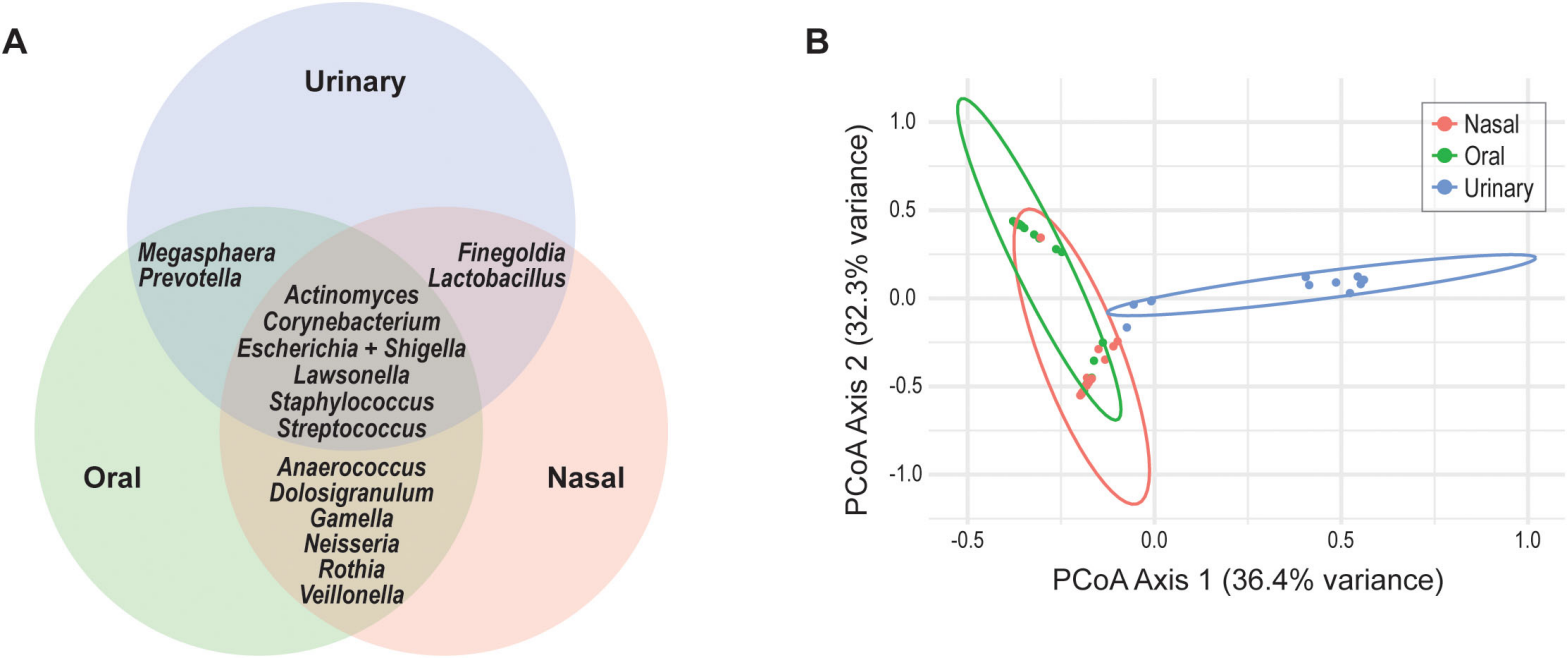
Compositional differences of the three anatomical site communities. **(A)** Genera found in the microbiomes of two or more anatomical sites. **(B)** PCoA plot assessing compositional differences between the nasal, oral, and urinary microbiomes.

Some of the genera identified were present in microbiomes from more than one anatomical site (**Figure 4A**). However, many of these taxa are not abundant in more than one microbiome (**Supplementary Table S2**). Statistical analysis revealed significant differences in genus-level presence and relative abundance across the three microbiome sites. Kruskal-Wallis test identified significant differences in relative abundance for several genera across sites. *Corynebacterium* (p = 0.000002), *Lawsonella* (p = 0.001426), and *Staphylococcus* (p = 0.000004) were significantly more abundant in the nasal microbiome, while *Streptococcus* (p = 0.000003) was enriched in the oral microbiome. As anticipated, *Lactobacillus* (p = 0.000000015) was predominantly abundant in the urinary microbiome.

Principal Coordinates Analysis (PCoA) based on Bray-Curtis dissimilarity was used to visualize differences in community composition across the three anatomical sites. As shown in **Figure 4B**, urine samples formed a distinct cluster, visually separating from both nasal and oral samples. This distinction was statistically supported by PERMANOVA (p = 0.001), confirming significant differences between the urine microbiome and the microbiomes of the other two sites. To further assess the compositional differences between nasal and oral microbiomes, pairwise PERMANOVA and ANOSIM analyses were performed. PERMANOVA revealed a significant difference between community compositions of the two sampled sites (F = 15.35, R² = 0.268, p = 0.001), indicating that site explained approximately 27% of the variance in microbial composition. ANOSIM results supported this finding, with a moderate degree of separation observed between nasal and oral samples (R^2^ = 0.43, p = 0.001).

## Discussion

While the nasal, oral, and urinary microbiomes of presumably healthy individuals have been studied largely in isolation (Costello et al., 2009; Bik et al., 2010; Human Microbiome Project Consortium, 2012; Bassis et al., 2014; Price et al., 2020a), comparatively little is known about how the urinary microbiome relates to more distant anatomical sites. Both the anterior nares and bladder are characterized as low-biomass microbial environments (Mueller et al., 2017; De Boeck et al., 2019). This is in contrast to the buccal microbiota, which exhibits an intermediate microbial biomass, albeit lower than that of other oral cavity sites including saliva, tongue dorsum, and dental plaque (Baker et al., 2024). Consistent with these biomass differences, profiling limitations persisted despite optimized extraction protocols and the use of sample-type–specific negative controls. Nevertheless, the genera identified across all sites align with those reported in prior studies of these respective microbiomes. Most microbiomes, regardless of the site sampled, are monodominated by a single genus, captured in the Simpson Diversity Index (**Figures 1**–**3**).

The urinary microbiome was typically monodominant, with *Lactobacillus* as the predominant genus, consistent with prior studies finding that this as the most common urotype (>50% relative abundance) across continent, asymptomatic females (Price et al., 2020a). Two individuals exhibited alternative urotypes: one predominated by *Escherichia* + *Shigella* (212U) and another by *Gardnerella* (218U). Previous work has shown that the *Gardnerella* urotype is more common in younger females, such as those sampled here, whereas the *Escherichia* urotype is more frequently observed in older, post-menopausal females (Price et al., 2020a). Although *Escherichia*, most notably *E. coli*, is commonly associated with UTI symptoms (Foxman, 2014), it is also frequently detected in the bladders of asymptomatic females (Thomas-White KJ. et al., 2018; Garretto et al., 2020; Price et al., 2020a). Four of the 26 samples represented mixed urotypes, with no single genus exceeding 50% relative abundance. Mixed urotypes have previously been associated with Black/African American females (Price et al., 2020a); however, because we did not collect this data from participants, we were unable to assess this association.

Of the six genera detected across the nasal, oral, and urinary microbiomes (**Figure 4A**), most are best characterized as transient or intermittently detected rather than core community members (**Supplementary Table S2**). *Streptococcus* is a recognized core member of the buccal microbiome (Mark Welch et al., 2019), and *Corynebacterium*, *Staphylococcus*, and *Streptococcus* constitute core taxa of the nasal microbiome (Frank et al., 2010; Blum et al., 2022). In contrast, the urinary microbiome lacks a core across females (Price et al., 2020c; Biehl et al., 2022). Consistent with these prior studies, our data show that *Actinomyces*, *Escherichia* + *Shigella*, and *Lawsonella* are infrequent, often low-abundance, genera in the oral and nasal microbiomes (**Figures 2**, **3**). Although *Corynebacterium*, *Staphylococcus*, and *Streptococcus* are abundant in the oral and nasal microbiomes of some individuals, they are never dominant in the urinary microbiome and are undetected in many samples.

Our study finds that the urinary microbiome is distinct from both the nasal and oral communities (**Figure 4B**). In contrast, the nasal and oral samples show overlapping distributions in Bray–Curtis PCoA space. While the microbiomes of these two sites are found to be different, supported by a p value <0.05, the effect size is moderate. The majority of variation (>70%) remained unexplained, consistent with the high inter-individual and stochastic variability typical of microbiome datasets.

Because we employed short-read 16S rRNA gene sequencing, species-level resolution was not possible. Nevertheless, even at the genus level—the highest resolution supported by our data—we find that the urinary microbiome is distinct from both the nasal and oral communities. Although our cross-sectional study did not identify evidence of any association between the urinary microbiome and the more distant sites investigated, future longitudinal analyses incorporating long-read 16S rRNA gene sequencing or shotgun metagenomics would be needed; this would enable researchers to determine whether the microbiome at one anatomical site is associated with that of another, as well as to discriminate between taxa that are transient from those that are persistent.

## Data Availability

The data presented in the study are deposited in the NCBI SRA repository, BioProject accession number PRJNA1246981. SRA accession numbers for each sample can be found in Supplementary Table 1.
