# Supplementary material for: Shared taxa but distinct communities: within-individual comparisons of oral, nasal, and urinary microbiomes in asymptomatic “healthy” females
Source: Front Microbiomes. 2026 Mar 9;5:1691965. doi: 10.3389/frmbi.2026.1691965 (PMC12993670; doi:10.3389/frmbi.2026.1691965)
Supplement: Supplementary file 1 [file DataSheet1.pdf]

S. epidermidis isolate	Isolation Source	No. of raw reads	SRA Accession No.
212U	Urine	277300	SRR33112688
213N	Nasal	280434	SRR33112687
213U	Urine	263800	SRR33112686
214N	Nasal	105072	SRR33112685
214O	Oral	297788	SRR33112684
214U	Urine	196008	SRR33112682
215N	Nasal	362394	SRR33112681
215O	Oral	358128	SRR33112680
215U	Urine	332630	SRR33112657
216N	Nasal	207884	SRR33112679
216O	Oral	263722	SRR33112668
216U	Urine	151234	SRR33112656
217N	Nasal	367956	SRR33112678
217O	Oral	249148	SRR33112667
217U	Urine	331448	SRR33112654
218N	Nasal	332394	SRR33112676
218O	Oral	307196	SRR33112665
218U	Urine	261522	SRR33112653
219N	Nasal	337558	SRR33112675
219O	Oral	326280	SRR33112664
219U	Urine	438312	SRR33112652
220N	Nasal	440378	SRR33112674
220O	Oral	315252	SRR33112663
220U	Urine	514682	SRR33112651
221N	Nasal	413442	SRR33112673
221O	Oral	451986	SRR33112662
221U	Urine	287676	SRR33112650
223N	Nasal	393738	SRR33112672
223O	Oral	303510	SRR33112661
223U	Urine	233154	SRR33112649
224N	Nasal	321602	SRR33112671
224O	Oral	287216	SRR33112660
224U	Urine	243626	SRR33112648
225N	Nasal	363136	SRR33112670
225O	Oral	348780	SRR33112659
225U	Urine	251644	SRR33112647
226N	Nasal	368138	SRR33112669

S. epidermidis isolate	Isolation Source	No. of raw reads	SRA Accession No.
226O	Oral	331154	SRR33112658
226U	Urine	402716	SRR33112646

Supplementary Table 1. Sample sequence information.
