# Supplementary material for: Shared taxa but distinct communities: within-individual comparisons of oral, nasal, and urinary microbiomes in asymptomatic “healthy” females
Source: Front Microbiomes. 2026 Mar 9;5:1691965. doi: 10.3389/frmbi.2026.1691965 (PMC12993670; doi:10.3389/frmbi.2026.1691965)
Supplement: Supplementary file 2 [file DataSheet2.pdf]

Genus	Oral – Mean (%)	Nasal – Mean (%)	Urine – Mean (%)
Streptococcus	68.8	10.4	< 1.0
Corynebacterium	12.2	46.8	< 0.3
Staphylococcus	2.6	28.5	1.0
Escherichia–Shigella	~1.0	< 0.1	4.96
Lawsonella	2.1	6.5	< 0.5
Actinomyces	1.1	2.5	< 0.5

Supplementary Table 2. Mean relative abundance for genera found in samples from all three anatomical sites.
